# Laparoscopic versus laparotomic surgery for adnexal masses: role in elderly

**DOI:** 10.1186/s12957-016-0861-1

**Published:** 2016-04-07

**Authors:** F. M. Pulcinelli, M. Schimberni, R. Marci, F. Bellati, D. Caserta

**Affiliations:** Department of Medical and Surgical Sciences and Translational Medicine, Sant’Andrea Hospital, Sapienza University of Rome, Via di Grottarossa, 1035/1039-00189 Rome, Italy; Department of Morphology, Surgery and Experimental Medicine, Sant’Anna University Hospital, Via Aldo Moro, 8-44124 Cona Ferrara, Italy

**Keywords:** Surgery in elderly, Adnexal masses, Laparoscopy in elderly, Laparotomy, In elderly

## Abstract

**Background:**

The purpose of this study is to compare laparoscopy (LPS) and laparotomy (LPT), in terms of surgical outcomes, in elderly patients (>65 years) with adnexal masses.

**Methods:**

We retrospectively reviewed a series of women older than 65 who had a diagnosis of adnexal masses. Then, all patients were divided into two different groups according to the type of surgery: 27 who underwent LPS (LPS group) and 24 who underwent LPT (LPT group). We took into consideration: age, comorbidity, histological diagnosis, surgery approach, and surgical outcome. Then, we calculated the percentages of all of these data and then *χ*^2^ test and *t*-Student test were used to calculate the *p* value, to compare the two surgical techniques. A *p* value lower than 0.05 was considered to be statistically significant.

**Results:**

At first, we evaluated the relation between the diagnosis and the surgery approach, and we obtained statistically significant results for serous cyst, adenocarcinoma serous/mucinous, and others, and the table highlights that some of the benign masses were mostly treated with LPS, while borderline and malignant masses were treated with LPT. Then, we evaluated the comorbidities of the patients, and we found that those cases had a significantly higher prevalence of cardiovascular disease and metabolic diseases. Finally, we compared the surgery outcome of LPS versus LPT surgeries for adnexal masses in elderly women, and there were statistically significant results for postoperative complications, number of patients who needed drainage, and number of days of hospitalization after surgery.

**Conclusions:**

Our results demonstrated that the patients who underwent LPS, compared to the patients who underwent LPT, have better outcomes in terms of postoperative complications (7.4 % with LPS and 37 % with LPT), number of patients who needed drainage (11.1 % with LPS and 62.5 % with LPT), and number of days of hospitalization after surgery, in term of mean (5 for LPS and 10.9 in term of LPT).

## Background

Adnexal masses are a common clinical problem that affect women of all ages. About 5–10 % of women in the USA run the risk of undergoing surgery for a suspected ovarian neoplasm, and 13–21 % of them have the chance of receiving a diagnosis of ovarian cancer [1].

These masses are frequently accidentally diagnosed, and they could be both gynecological and non-gynecological, and they can represent a benign, borderline, or malignant neoplasm, metastasis from distant site, or a non-neoplastic process [2].

For this reason, the management of adnexal mass remains controversial. Due to the uncertainty of most lesions, the best surgical approach is still debated. The risk of performing an extended surgery, as laparotomy (LPT), for benign disease, or the risk of performing a minimally invasive surgery, as laparoscopy (LPS), for malign disease [3], still remains.

The elderly population has an increased risk of morbidity like cardiovascular disease, diabetes mellitus, and obesity, and when an elderly patient, who needs surgery, suffers from some of these diseases, surgical morbidity and mortality rates increase. In fact, according to literature, higher age at the time of surgery is associated with a worst prognosis [4].

Due to the increase in life expectancy, it is expected that in 2030, 20 % of the US population will be older than 65 making the treatment of adnexal masses in elderly patients more frequent, so it means that this percentage will increase in the future [5].

Thus, it seems important to establish risk profiles of all patients with adnexal masses so they can reap the benefit of minimally invasive surgery wherever possible.

Laparoscopic surgery has been progressively integrated into standard adnexal mass care in the past years.

Despite the well-known advantages of LPS, it is today still contraindicated for patients with multiple comorbidities such as the ones described before.

In fact, age, cardiovascular disease, and obesity are relative contraindications to LPS. That is because for these patients, it is difficult to establish pneumo-peritoneum, ventilation, and they are unable to tolerate the Trendelenburg position [6].

According to literature, old age at the time of surgery is associated with a worst prognosis.

So, a less-invasive surgical approach appears to be the best choice among this group of patients, which allows shorter stay and lower postoperative morbidity [7].

So, on one hand there are authors who say that elderly patients, with comorbidities described, are not able to tolerate a LPS, and on the other hand there are others who say that a shorter surgery may reduce postoperative complications and days of hospitalization after surgery, which seemed to worsen pre-existing conditions of the patients.

The purpose of this study is to compare LPS to LPT in elderly patients, >65 years, with adnexal masses in terms of morbidity, mortality, and surgery outcome.

## Methods

We retrospectively reviewed a series of consecutive women older than 65 years who had a diagnosis of adnexal mass.

The diagnosis of such women was carried out by clinical signs and symptoms, transvaginal ultrasonography (US), Color Doppler US, the dosage of serum markers, and MRI/CT scan when indicated, to characterize if the mass had benign or malignant aspects.

We analyzed and collected data from 51 patients who underwent surgery for adnexal masses in the Unit of Gynecologic-Obstetrical of Sant’Andrea Hospital, Sapienza University of Rome.

Patients were divided into two different groups according to the type of surgery: 27 subjected to LPS (LPS group) and 24 subjected to LPT (LPT group).

We took into consideration:

- Age;
- Comorbidity, in terms of how many patients had underlying conditions, we know that by their history and then we evaluated the surgical approach for each of these patients (Tables 2, 3);
- Histological diagnosis;
- Surgical approach; and
- Surgical outcome in terms of postoperative complications, postoperative bleeding, number of patients who needed drainage, number of days that such patients maintained drainage, number of days before analyzing again, and number of days of hospitalization after surgery.

*χ*^2^ test and *t*-Student test were used where appropriate to calculate the *p* value, to compare the two surgical approaches. A *p* value lower than 0.05 was considered to be statistically significant.

## Results

Our study group consisted of 51 women subjected to surgery from 2009 to 2014. The mean age was 72.9 (range 65–88 years). The mean age of the LPS group was 71.4 (65–81 years) compared to the LPT group 74.6 (range 65–88 years).

We found the following masses in patients undergoing both LPS and LPT surgery: endometriosis, serous cyst, corpus luteum, fibroma, teratoma, cystadenoma serous/mucinous, stromal ovarian tumors, adenocarcinoma serous/mucinous, borderline, and others (Table 1).

**Table 1:** Relation between the diagnosis and the surgery approach

Nature of the mass	LPS		LPT		*p*
	Number	%	Number	%	
Endometriosis	1	2.4	0	0	NS
Serous cyst	18	43.9	5	19.2	0.0382
Fibroma	6	14.6	2	7.7	NS
Teratoma	0	0	2	7.7	NS
Cystadenoma serous/mucinous	6	14.6	2	7.7	NS
Stromal ovarian tumors	0	0	2	7.7	NS
Germinal ovarian tumors	0	0	1	3.8	NS
Adenocarcinoma serous/mucinous	1	2.4	9	34.6	0.0003
Borderline	0	0	2	7.7	NS
Others	9	21.9	1	3.8	0.0427
Total	41		26		

*NS* non significant: *p* > 0.05

As for the rupture of the corpus luteum, fibroma, teratoma, cystadenoma serous/mucinous, and stromal ovarian tumors, the difference between the two groups LPS and LPT is not statistically significant. While the statistically significant results are about serous cysts, adenocarcinoma serous/mucinous, and others, this table highlights that some of the benign masses were mostly treated with LPS, while borderline and malignant masses were treated with LPT.

Surgeries that we performed with the LPS approach were salpingo-oophorectomy, oophorectomy, salpingectomy, and cystectomy.

Surgeries performed with the LPT approach were hysterectomy, salpingo-oophorectomy, oophorectomy, salpingectomy, cystectomy, lymphadenectomy, omentectomy, appendicectomy, and colorectal resection.

Then, we evaluated the comorbidities of the patients and we found that those cases had a significant higher prevalence of cardiovascular disease (*p* = 0.0001) and metabolic diseases (*p* = 0.0214) (Table 2).

**Table 2:** Comorbidity in two groups

Comorbidity	LPS		LPT		*p*
	Number	%	Number	%	
Cardiovascular disease	3	10.7	24	66.7	<0.0001
Neurological disease	3	10.7	4	11.1	NS
Thyroid disease	1	3.6	1	2.8	NS
Lung disease	1	3.6	2	5.6	NS
DM	0	0	2	5.6	NS
Gastro-intestinal disease	1	3.6	3	8.3	NS
Metabolic disease	19	67.9	14	38.9	0.0214
Total	28		36		

*NS* non significant: *p* > 0.05

These tables show that in cardiovascular diseases and metabolic diseases, there is a statistical significance, with greater numbers of patients suffering from these diseases in the LPT group.

Preoperative comorbidities, the disease that we found are described in Table 3.

**Table 3:** All diagnoses of each comorbidity in the two groups

Comorbidity	LPS	LPT
Cardiovascular disease		
-Hypertension	15	17
-Heart failure	0	2
-Atrial fibrillation	1	2
-Previous acute myocardial infarction	1	2
Neurological disease		
-Transient ischemic attack	2	3
-Parkinson’s disease	1	0
-Previous subarachnoid hemorrhage	1	1
Thyroid disease		
-Hypothyroidism	1	0
-Multinodular goiter	0	1
Lung disease		
-Chronic obstructive pulmonary disease.	1	2
Diabetes mellitus	0	2
Gastro-intestinal disease		
-Ascites	0	2
-Hepatitis B	1	0
-Colon carcinoma	0	1
Metabolic diseases		
-Overweight	3	2
-Obese	7	7
-Hypercholesterolemia	9	5

The elderly population had an increased risk of morbidity like cardiovascular disease, diabetes mellitus, and obesity, and when an elderly patient, that needs surgery, suffers from some of these diseases, surgical morbidity and mortality rates increase [4].

So, it is important to establish risk profiles of all patients with adnexal masses so they can reap the benefit of minimally invasive surgery wherever possible.

Despite the well-known advantages of LPS, it is today still contraindicated for patients with comorbidities that we described before. On the one hand, there are authors who say that elderly patients, with the comorbidities described, are not able to tolerate a LPS; in fact, age, cardiovascular disease, and obesity are relative contraindications to LPS.

So, the surgeons preferred to operate on these patients with the LPT approach, because when an elderly patient, that needs surgery, suffers from some of these diseases, surgical morbidity and mortality rates increase [6], and on the other hand, there are authors who say that a shorter, and less-invasive surgery may reduce postoperative complications and days of hospitalization after surgery.

For this reason, we compared the surgery outcome of LPS versus LPT surgeries for adnexal masses in elderly women (Table 4).

**Table 4:** Surgical outcome

Surgical outcome	LPS (27)		LPT (24)		*p*
	Number	%	Number	%	
Postoperative complications	2	7.4	9	37.5	0.0091
Postoperative bleeding	1	3.7	1	4.2	NS
Drainage number of patient	3	11.1	15	62.5	0.0001
Drainage days (mean)	2	/	4.9	/	NS
Recanalization (mean)	2.9	/	5.1	/	NS
Days of hospitalization (mean)	5	/	10.9	/	0.0385

*NS* non significant: *p* > 0.05

Our study has some limitations. The number of patients who undewent surgery, in the 5 years, is not high: furthermore, we wanted to follow the patients with a long-term follow-up, but this was not possible because only a minority of these patients returned to the medical examination after a few months.

This study has, also, strengths. The number of patients examined, although it was not high, was sufficient to have a good series, although not all surgical outcomes had a *p* value lower than 0.05, those who came statistically significant were important to demonstrate that the patients who underwent LPS surgery, compared to the patients who underwent LPT, had better outcomes, even if some authors prefer the LPT for patients with comorbidities.

Comparing LPS and LPT, our results showed that there were statistically significant results for postoperative complications, number of patients who needed drainage, and number of days of hospitalization after surgery (Table 4).

Postoperative complications were mainly caused by infections.

As for LPS, we had two patients with high fever for several days, treated with antibiotics.

As regards LPT, we had six patients with high fever for several days treated with antibiotics, two infections of the surgical sutures, one patient had a bowel obstruction, for which she underwent a second surgery; she also had pneumonia and a pancreatitis.

## Discussion

We evaluated the relation between the diagnosis and the surgery approach. We found the following masses in patients undergoing both LPS and LPT surgery: endometriosis, serous cyst, corpus luteum, fibroma, teratoma, cystadenoma serous/mucinous, stromal ovarian tumors, adenocarcinoma serous/mucinous, borderline, and others.

We obtained statistically significant results for serous cyst, adenocarcinoma serous/mucinous, and others, and the table highlights that some of the benign masses were mostly treated with LPS, while borderline and malignant masses were treated with LPT.

Mohamad S. Gad and James H. Liu, in their paper, said that LPS was used mainly for benign masses, instead LPT was used mainly for malinant and borderline masses, these results are comparable to our results [8, 9].

As for Table 3, we evaluated the comorbidities of the patients and we found that cases had a significant higher prevalence of cardiovascular (*p* = 0.0001) and metabolic diseases (*p* = 0.0214).

The table shows that, for what concerns cardiovascular diseases and metabolic diseases, there is a statistical significance, with greater numbers of patients suffering from these diseases in LPT group.

In Table 4, we compared the surgery outcome of LPS versus LPT surgeries for adnexal masses in elderly women. Comparing LPS and LPT, there were statistically significant results for postoperative complications, number of patients who needed drainage, and number of days of hospitalization after surgery.

The elderly population has an increased risk of morbidity like cardiovascular diseases, diabetes mellitus, and obesity, and when an elderly patient that needs surgery, suffers from some of these diseases, surgical morbidity and mortality rates increase [4].

So, it is important to establish the risk profiles of all patients with adnexal masses so they can reap the benefit of minimally invasive surgery whenever possible.

Laparoscopic surgery has been progressively integrated into standard adnexal massecare during the past years.

Despite the well-known advantages of LPS, today it is still contraindicated for patients with comorbidities that we described before.

In fact, age, cardiovascular diseases, and obesity are relative contraindications to LPS. This is because it is difficult to establish pneumo-peritoneum, ventilation for such patients, and they are unable to tolerate the Trendelenburg position [6].

Nevertheless, surgery is the most used method to treat the adnexal masses and the complexity of treatment of elderly patients; it is the result of a large number of comorbidities and, as a consequence, the potential possibility of a large number of complications, especially postoperative ones.

According to literature, higher age at the time of surgeries, associated with a worse prognosis is not recommended [10].

So, a less-invasive surgical approach appears to be the best choice in this group of patients, as it allows a shorter stay and lower postoperative morbidity, which seemed to worsen pre-existing conditions of the patients [7].

In fact, postoperative complication is associated with morbidity, decreased quality of life, delay in receipt of chemotherapy, and mortality [11].

So, on the one hand, there are authors who say that elderly patients with the comorbidities described are not able to tolerate a LPS treatment, and on the other hand, there are authors who say that a shorter and less-invasive surgery may reduce postoperative complications and days of hospitalization after surgery, which seemed to worsen pre-existing conditions of the patients.

Thus, our results demonstrated that the patients who underwent LPS, compared to patients who underwent LPT, have better outcomes in terms of: postoperative complications (7.4 % with LPS and 37 % with LPT), number of patients who needed drainage (11.1 % with LPS and 62.5 % with LPT), and number of days of hospitalization after surgery, in term of mean (5 for LPS and 10.9 in term of LPT) (Table 4).

So, despite some authors who say that the LPT may be safer, especially for obese patients, and/or with cardio vascular diseases, our results show that patients with these comorbidities, who underwent LPS, have a better outcome, this seems to reduce the risk of worsening pre-existing conditions of the patients.

## Conclusions

On the one hand there are authors who say that elderly patients with the comorbidities described are not able to tolerate a LPS, in fact, age, cardiovascular disease, and obesity are relative contraindications to LPS. This is because for these patients, it is difficult to establish pneumo-peritoneum, ventilation, and they are unable to tolerate the Trendelenburg position.

On the other hand, there are authors who say that a shorter, and less-invasive surgery may reduce postoperative complications and days of hospitalization after surgery, which seemed to worsen the pre-existing conditions of the patients.

According to literature, older age at the time of surgery is associated with a worst prognosis [10]; nevertheless, surgery is the best method to treat the adnexal masses, and the complexity of treatment of elderly patients is a result of a large number of comorbidities and, as a consequence, the potential possibility of a large number of complications, especially postoperative complications.

For this reason, we compared the surgery outcome of LPS versus LPT surgeries for adnexal masses in elderly women.

Our results demonstrated that patients who underwent LPS surgery, compared to patients underwent LPT, had better outcomes in terms of: postoperative complications, number of patients who needed drainage, and number of days of hospitalization after surgery.

Our results show that in elderly patients with multiple comorbidities, laparoscopy remains the best treatment.

### Ethics approval and consent to participate

Not applicable.

### Consent for publication

Not applicable.

## Abbreviations

LPS: laparoscopy
LPT: laparotomy
